# Full-length transcriptome from different life stages of cobia (*Rachycentron canadum*, Rachycentridae)

**DOI:** 10.1038/s41597-022-01907-0

**Published:** 2023-02-16

**Authors:** Sanal Ebeneezar, S. R. Krupesha Sharma, P. Vijayagopal, Wilson Sebastian, K. A. Sajina, G. Tamilmani, M. Sakthivel, P. Rameshkumar, K. K. Anikuttan, Eldho Varghese, D. Linga Prabu, N. S. Jeena, T. G. Sumithra, S. Gayathri, G. Iyyapparaja Narasimapallavan, A. Gopalakrishnan

**Affiliations:** 1 Marine Biotechnology Fish Nutrition and Health Division, ICAR- Central Marine Fisheries Research Institute, Kochi, Kerala 682018 India; 2 Mandapam Regional Centre of ICAR- Central Marine Fisheries Research Institute, Mandapam Camp, Tamil Nadu 623520 India; 3 Tuticorin Regional Station of ICAR- Central Marine Fisheries Research Institute, Thoothukudi, Tamil Nadu 628001 India

**Keywords:** Transcriptomics, Biotechnology

## Abstract

Cobia (*Rachycentron canadum*, Rachycentridae) is one of the prospective species for mariculture. The transcriptome-based study on cobia was hampered by an inadequate reference genome and a lack of full-length cDNAs. We used a long-read based sequencing technology (PacBio Sequel II Iso-Seq3 SMRT) to obtain complete transcriptome sequences from larvae, juveniles, and various tissues of adult cobia, and a single SMRTcell generated 99 gigabytes of data and 51,205,946,694 bases. A total of 8609435, 7441673 and 9140164 subreads were generated from the larval, juvenile, and adult sample pools, with mean sub-read lengths of 2109.9, 1988.2 and 1996.2 bp, respectively. All samples were combined to increase transcript recovery and clustered into 35661 high-quality reads. This is the first report on a full-length transcriptome from *R. canadum*. Our results illustrate a significant increase in the identified amount of cobia LncRNAs and alternatively spliced transcripts, which will help improve genome annotation. Furthermore, this information will be beneficial for nutrigenomics and functional studies on cobia and other commercially important mariculture species.

## Abstract (summary)

**Table:** 

Measurement(s)	Full length transcriptome of Cobia (Rachycentron canadum)
Technology Type(s)	PacBio Sequel 2
Sample Characteristic - Organism	Rachycentron canadum
Sample Characteristic - Environment	Marine environment
Sample Characteristic - Location	India

## Background & Summary

With an annual growth rate of 5.8%, aquaculture is one of the most promising sectors of food production worldwide. World aquaculture production was 82 million tons in 2018, of which 54.3 million tons were contributed by finfish aquaculture^1^. Marine aquaculture has the potential to meet the increasing global demand for animal protein-based foods. Cobia (*R. canadum*), the only extant species in the family Rachycentridae, is a marine warm-water species distributed worldwide, particularly in tropical and subtropical climates, except for the central and eastern Pacific. In recent decades, cobia emerged as one of the most promising species for mariculture due to certain attributes like rapid growth rate, good meat quality and high market value, with a global production of around 40,000 tons^2–5^. In India, cobia was first successfully bred in 2010 at the Mandapam Regional Centre of the Central Marine Fisheries Research Institute in Tamil Nadu^6–8^. However, cobia aquaculture has been hampered by the deficiency of nutritional information, which limits the productivity of industrial forms of aquaculture^9^.

In order to optimise the efficient culture system of a species, we need to address the fundamental knowledge gap related to aspects of culture such as reproductive biology, digestive physiology and nutritional genetics^10^. To fill such a knowledge gap, an integrative study using different techniques is needed. Next-generation sequencing (NGS) studies can holistically elucidate the structures and functions of genes, as well as the molecular mechanisms underlying biological processes such as growth, nutrition, metabolism, immune function, stress, adaptation, and differential gene expression in response to factors such as diet, stress and other environmental factors^11–13^. Data from such systems has aided in the production of several commercially important fishes such as Chinese seabass (*Lateolabrax maculatus*^14^); Atlantic salmon (*Salmo salar*^15^), and Rainbow trout (*Oncorhychus mykiss*^16^). Thus, this information can be used to develop nutritional markers for different developmental stages and optimised feeding protocols^17^. For example, the effects of selected nutrients on target genes can be studied to adjust diet composition to improve growth, condition, and survival of fish larvae^10,18,19^. The identification of potential genes involved in key pathways involved in carbohydrate, lipid, amino acid, nucleotide, cofactor and vitamin metabolism will aid in the formulation of species and stage–specific diets for commercially important mariculture species such as cobia. However, previous studies on transcriptome analysis in cobia used short-read based platforms and were limited to a few tissue types^17,20–22^. Discovering novel transcripts, supporting genome annotation and identifying alternative splices and gene fusions require full-length transcripts, and as such, genetic data on cobia remain insufficient, limiting the scope of such research.

The full-length protein coding transcriptome of a species (including CDS and 5′ - and 3′ - UTRs) and its collection of splice variants are a crucial resource for the accurate annotation of protein-coding transcripts and for understanding how structural variants affect nutritional status, health, and economically significant traits in livestock^23,24^. Although next-generation short-read based sequencing has numerous advantages—for instance low cost, quantifiability and high throughput—it is less effective for assembling full-length transcripts with short sequencing runs without a reference genome, which could lead to inappropriate annotations^25,26^. The scope of studying alternative splice variants and corrected annotations is limited by low-quality transcripts attained by Illumina sequencing^27^. The most advanced third-generation sequencing platform (TGS) can aid us obtain a long-read or full-length transcriptome without assembly to study the structure of mRNAs, allowing us to discover more genes, detect alternative splicing, polyadenylation as well as long non-coding RNAs (LncRNAs)^28,29^. The TGS platforms have recently emerged as new genomic research tools owing to the advent of high-throughput sequencing technology.

The present study aims to generate full-length transcriptome for the commercially important mariculture fish, *R. canadum*, by sequencing individuals from different life stages using a TGS platform. The information generated from this research could be used to complement the genome for discovering new genes, gaining knowledge on the physiological properties and structure of mRNAs as well as for identifying potential nutritional markers in cobia.

## Methods

### Sample collection, preservation and RNA preparation

The animal experimental methods in this study were performed according to the ARRIVE recommendations^30^. The live fish were treated in accordance with the UK legislation: Animals (Scientific Procedures) Act (1986) of the United Kingdom (https://www.legislation.gov.uk/ukpga/1986/14/contents) and EU Directive on animal studies,2010/63/EU (2019)^31^. The experimental protocols used to conduct this study were approved by the ICAR-CMFRI, Kochi, India (BT/AAQ/3/SP28267/2018).

**Fig. 1:**
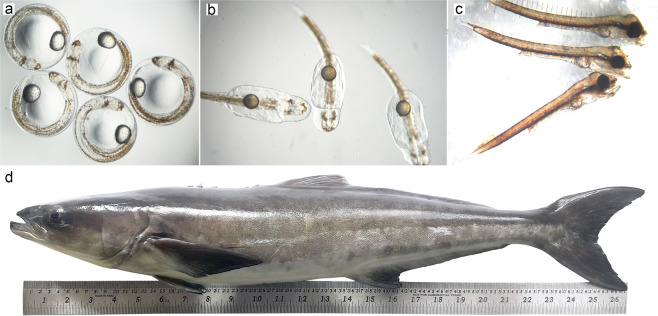
Different life-stages of cobia (*R. canadum*); **(a)** Embryo development. **(b)** Newly hatched larvae. **(c)** 3 dph larvae showing mouth opening. **(d)** Sub-adult.

The different life stages of the cobia are depicted in Fig. 1. *R. canadum* larval samples were collected from the Marine Fish Hatchery at the Mandapam Regional Centre of ICAR-Central Marine Fisheries Research Institute, India. The juvenile and adult samples were collected from the fish maintained in the high-density polyethylene sea cages (6 m diameter, 4 m depth, 113 m^3^) at Mandapam, Tamil Nadu, India (site 9 16′ 11.9748” N, 79 7′ 56.0856” E; Lat-Long = 9.269993, 79.132246). For larvae samples, a weight of 500–800 mg (contained around 300 larvae of 5 dph and 15 larvae of 29 dph) was collected in triplicates and then immediately stored in RNA protection reagent (RNAlater, Sigma-Aldrich) at −80 °C until RNA extraction. Also, tissue samples (muscle, kidney, spleen, liver, intestine, 500–800 mg each) from 3 individuals of juvenile and adult fish were collected and immediately stored in RNA protection reagent and maintained at −80 °C until RNA extraction.

Total RNA from each sample was extracted using the lithium chloride approach^32^ and purified using the NucleoSpin RNA clean-up kit (MACHEREY NAGEL) following the manufacturer’s protocol. After isolation, the RNA samples were analysed for quantity and integrity using the Qubit 4.0 fluorometer (ThemoFisher Scientific, USA) and the AGILENT Bio-analyser 2100 (Agilent, USA).

### PacBio Sequel Iso-seq3 library preparation and single molecule real-time (SMRT) sequencing

The RNA samples were divided into three pools prior to library construction. Pool 1 comprised whole larvae (5 and 29 days after hatching), Pool 2 comprised tissue samples from juveniles (muscle, kidney, spleen, liver, intestine) while Pool 3 comprised tissue samples from adults (muscle, pyloric caeca, spleen, intestine, kidney, liver). Equal amounts of RNA from each tissue were pooled to construct cDNA library.

Three Iso-Seq sequencing libraries were generated following the PacBio’s Iso-Seq3 protocol. Briefly, 2 µg of purified polyA mRNA was reverse transcribed into cDNA using the NEBNext Single Cell/Low input cDNA synthesis, while the second strand was synthesized by template switching. The cDNA preparation was purified using Pronex beads (Promega) and the purified cDNA was PCR amplified and repurified using specific Pronex beads to obtain standard transcripts, and analysed in the Bioanalyzer (Agilent Technologies, USA). After size selection using the BluePippin^TM^ size selection system, DNA damage repair and terminal repair were performed on the SMRTbell libraries, followed by overhand adapter ligation, and equimolar amounts of the barcoded cDNA were pooled. A quantity of 132 ng HiFi SMRTbell libraries was prepared with a final concentration of 13.2 ng of purified cDNA. After polymerase binding and primer annealing with PacBio sequencing primers on SMRT templates, the SMRTbell containing 60 pM OPLC-purified polymerase-bound SMRTbell complex was finally processed for sequencing on the PacBio Sequel II platform at Nucleome Informatics (P) Ltd., Hyderabad, India.

### The output of PacBio Sequel II sequencing and error rectifications

Three multiple tissue libraries were sequenced on the PacBio Sequel II platform and a total of 99 GB data was generated, mean sub-read lengths including 2109.9 bp, 1988.2 bp, and 1996.2 for larval (Pool 1), juvenile (Pool 2), and adult (Pool 3) sample pools, respectively (Table 1).

**Table 1:** Details of RNA sample pooling and PacBio Iso-seq output statistics.

Details of RNA samples pooling			
Pool	Description	Life stage	Group
Pool 1	Whole Cobia larvae of 5 dph and 29 dph	Larvae	Pool 1
Pool 2	Muscle, kidney, spleen, liver, intestine	Juveniles	Pool 2
Pool 3	Muscle, pyloric caeca, spleen, intestine, kidney, liver	Adults	Pool 3
PacBio Iso-seq output statistics			
Libraries	Pool 1	Pool 2	Pool 3
Subreads	8609435	7441673	9140164
Total bases	18164983812	14795758862	18245204020
Mean of Longest Subread Length	2109.9	1988.2	1996.2
Number of circular consensus sequence reads (CCS)	219158	179015	222843
Mean length of CCSs	2404.9	2188.2	2258.9
Total bases of CCSs	527057778	391715180	503380988
Number of full length reads	8609435	7441673	9140164
Number of full length non chimeric reads	4517416	3900355	4791753
Number of full length non chimeric reads with poly-A	4079227	3528745	4330583
Non-redundant isoforms			
Total number	35661		
Total bases	94193725		
Maximum length	11372		
Minimum length	181		
N50	2984		

Further analysis revealed 219,072, 178,893 and 222,754 full-length non-chimeric reads (FLNC) for sample from Pool 1, Pool 2 and Pool 3, respectively. FLNC from all samples were combined to increase transcript recovery and resulted in 35661 high-quality, non-redundant isoform sequence sets with a total of 94193725 nucleotide bases, while the mean length of transcripts was 3110 bp, and N50 value was 2984 bp (Table 1).

### Sequence data analysis

The raw data generated with the PacBio Sequel platform was analysed and processed using the standard protocol in SMRT Link software, while subreads were obtained by removing the adapters from the sequences and sorting out the polymerase reads with fragment lengths less than 50 bp, having a quality of 0.90. Meanwhile, subreads with a length of less than 50 bp were discarded, and the remaining subreads represented clean data. Circular consensus sequences (CCS) with full passes of ≥ 1 and a quality of > 0.90 were retrieved from the clean data, and by determining the presence of sequencing primers and terminal polyA sequence, the CCS were categorised into full-length nonchimeric CCSs and non-full-length nonchimeric CCSs. The presence of 5′ adapter, 3′ adapter sequence and poly A tails in the sequences was used to determine full-length non-chimeric readings (FLNC). Isoseq 3 software was used to extract and polish consensus isoforms in FLNC. The criterion for achieving high-quality, full-length transcripts was >99% post-correction accuracy. The CD-HIT software^33^ was used to eliminate redundant sequences from high-quality, full-length transcripts, and the full-length transcriptome from this step was used as the final isoform set of non-redundant transcripts used for further analysis. TransDecoder v3.0.1 software (TransDecoder. https://transdecoder.github.io/) was used to envisage the open reading frames (ORFs) of the non-redundant transcript isoform set with the lowest CDS of 100 bp. Finally, transcriptome completeness was analysed using the Benchmarking Universal Single Copy Orthologs (BUSCO) analysis^34^ based on the Ortholog database v9^35^.

### Functional annotation of full-length transcriptome

Full-length transcripts were annotated by BLASTx and BLASTp searches against NCBInr (http://www.ncbi.nlm.nih.gov), RefSeq^36^, UniProtKB, KOG (http://www.expasy.ch/sprot, version: 2019-8-14) and Pfam (v26.0) databases with an E-value cut off of 1e-5^37^. We found one best match among each transcript and a known sequence in the database based on bit score. Metascape^38^ and EggNOG^39^ analyses were performed for Gene Ontology (GO) annotation, and to classify the function of the transcript based on cellular components, molecular functions and biological process features. To obtain the overall biological function of *R. canadum* transcriptome, the full-length transcripts were mapped into canonical reference pathways in KEGG using KEGG KASS^40^, while the TransDecoder v3.0.1 software was employed to find functional protein domains and to predict the ORFs of the non-redundant transcripts.

Annotation of the transcriptome with several databases (NCBI nr, RefSeq and UniprotKB) revealed in a functional assignment for 19081 transcripts (53.51%). Most sequence similarities were against the NCBI nr. (34783 transcripts, 97.54%), followed by the UniprotKB database (33321 transcripts, 93.44%), the Pfam database (32888, 92.22%) and the RefSeq database (19081 transcripts, 53.5%) (Table 2). In the NCBI nr annotation, 11948 (34.35%) of the homologous sequence was aligned to *Seriola dumerili*, followed by *Seriola lalandi* dorsalis (5895, 16.95%), *Echeneis naucrates* (4697, 13.50%), and *Lates calcarifer* (4063, 11.68%).

**Table 2:** Annotation statistics.

Database	Full-length transcripts of Cobia	%
NCBI nr	34783	97.55
RefSeq	19081	53.51
UniProtKBSwiss_Prot	33321	93.44
GO annotation	21322	59.79
Pfam	32888	92.22
KEGG KO	26893	75.41
KOG	34219	95.96
Annotated in all 7 databases	7736	21.69
Annotated in at least 1 database	35526	99.62
Not Annotated in any database	135	0.38

**Fig. 2:**
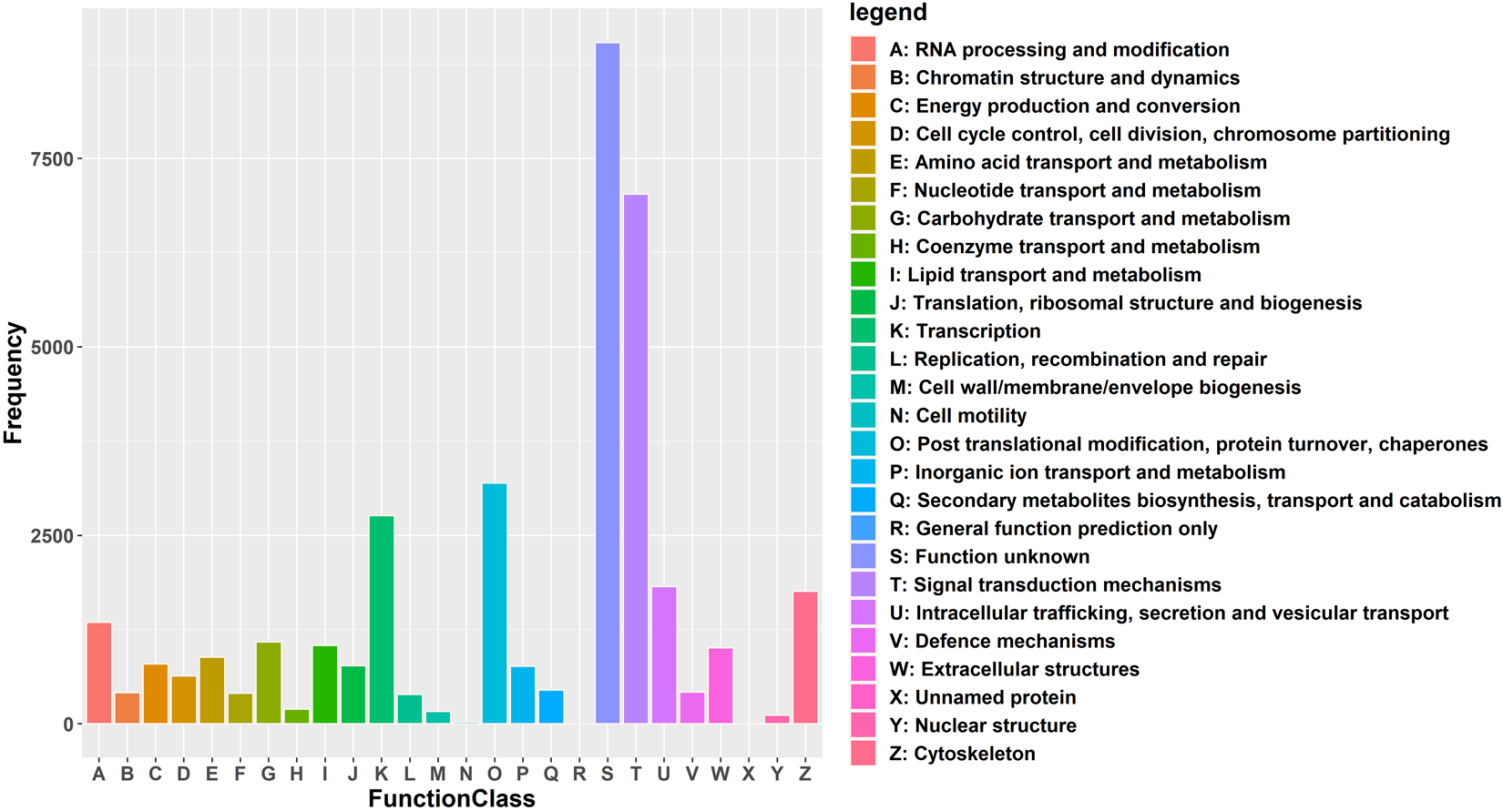
KOG function classification of transcript of *R. canadum*. x- axis represent different KOG categories (denoted by legends on right), and the y- axis, the number of the transcripts.

**Fig. 3:**
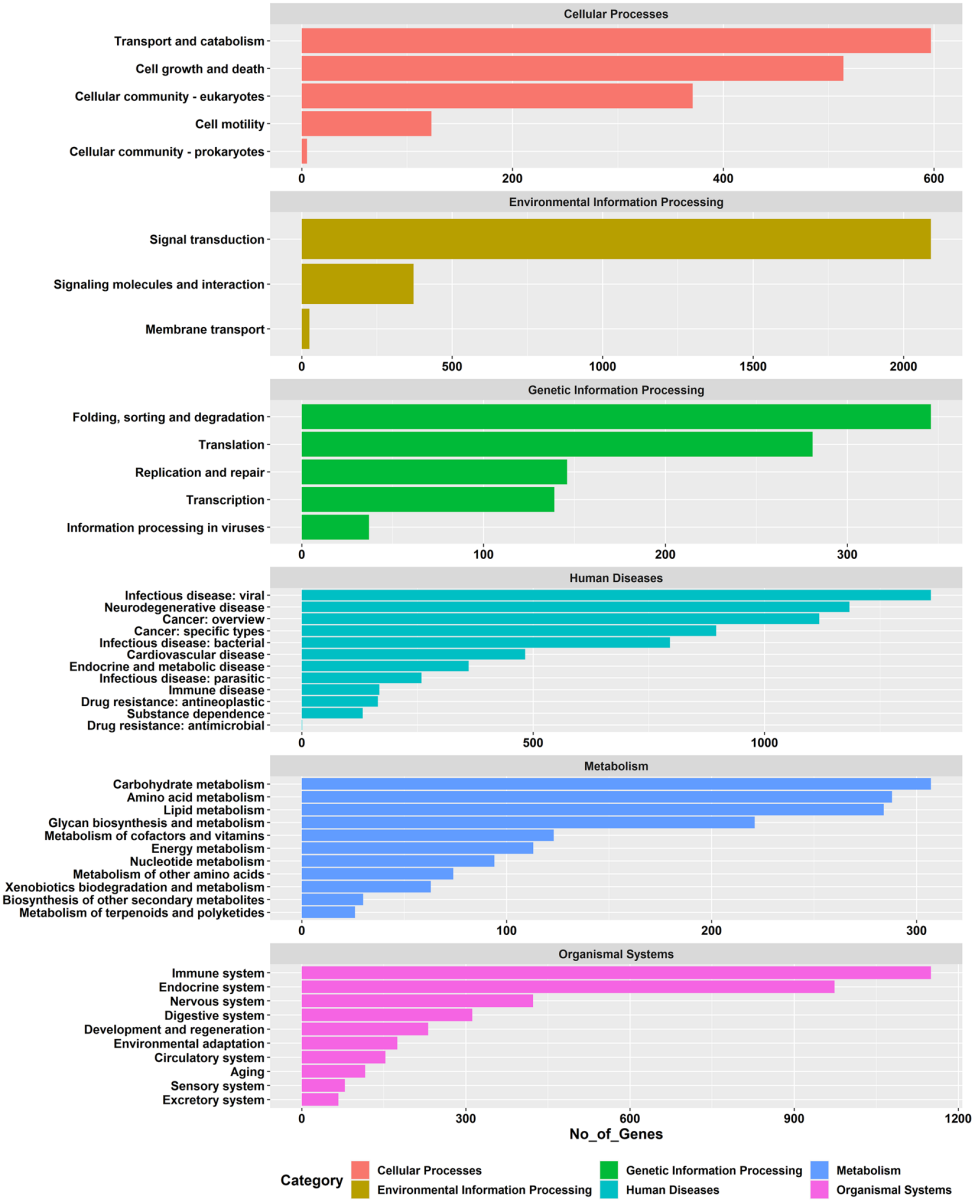
Identified KEGG pathways of transcript isoforms of *R. canadum*. The x-axis signifies the number of genes, and the y-axis, different KEGG pathways.

The KOG-annotated transcripts were grouped into 26 KOG classifications, with the highest number of transcripts in the function unknown category (S) (9038, 24.75%), signal transduction mechanism (7026, 19.24%) followed by posttranslational modification, protein turnover, chaperones (3196, 8.75%), transcription (2765, 7.57%) and intracellular trafficking, secretion, and vesicular transport (1822, 4.99%) (Fig. 2). For KEGG annotation, transcripts were mainly grouped into 398 signalling pathways in 48 level 2 pathways, among which, the signal transduction pathway (T) had the highest number of transcripts (2091), followed by infectious diseases- viral (1360) and immune system (1150) (Fig. 3).

**Fig. 4:**
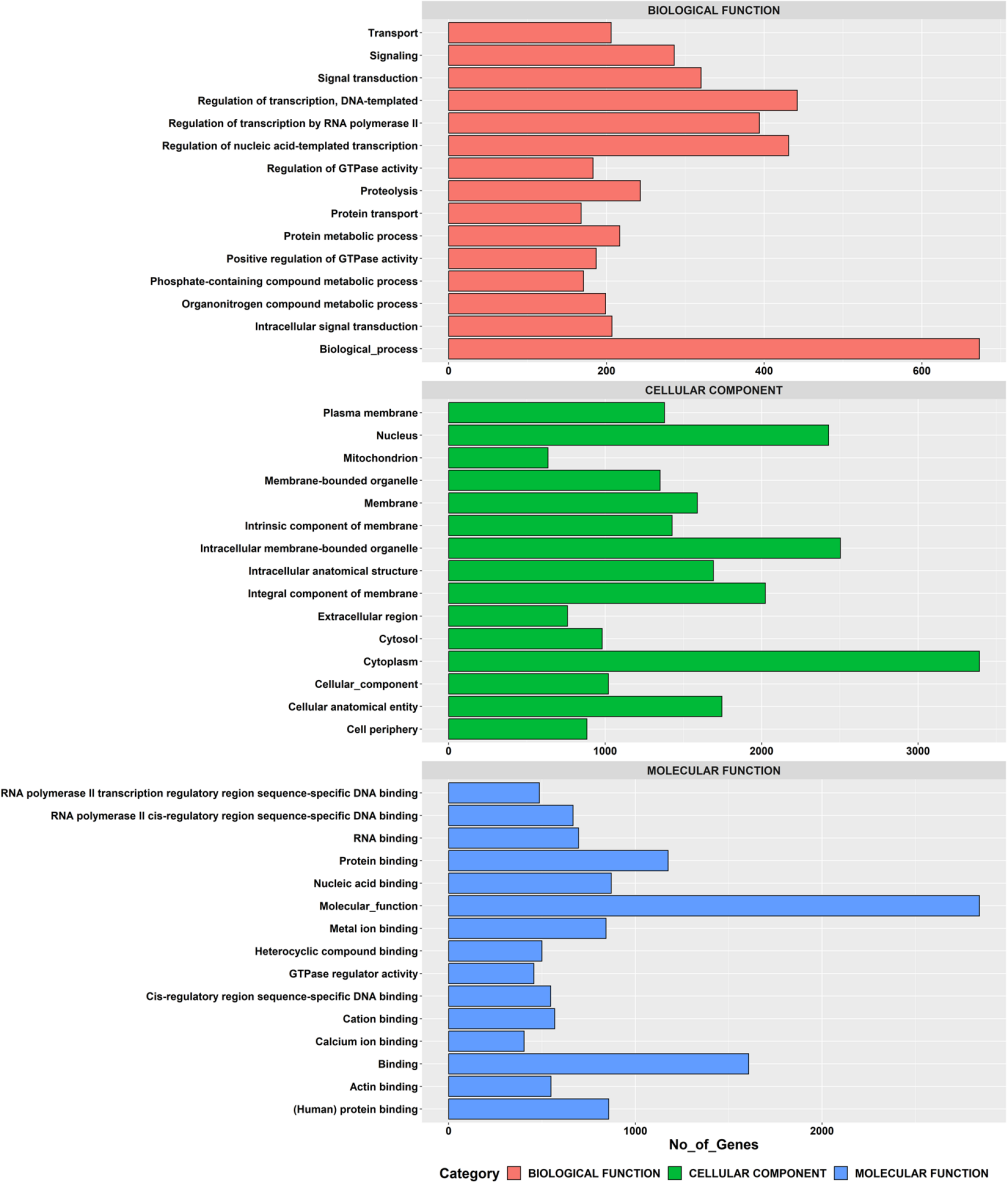
Gene function classification of *R. canadum* based on Gene ontology (GO) annotation. The x-axis denotes the number of genes, and the y-axis- different GO categories.

After GO annotation, a total of 20975 (58.82%) transcripts were allocated to multiple GO terms, among which 6829 transcripts (19.15%) were allotted to biological process, 3057 transcripts (8.57%) to molecular function and 1533 transcripts (4.30%) to cellular component (Fig. 4). Of all transcripts, 35526 (99.62%) were successfully annotated in at least one database and 7736 (21.69%) were annotated in all databases (Table 2).

### Genes related to nutrition

**Fig. 5:**
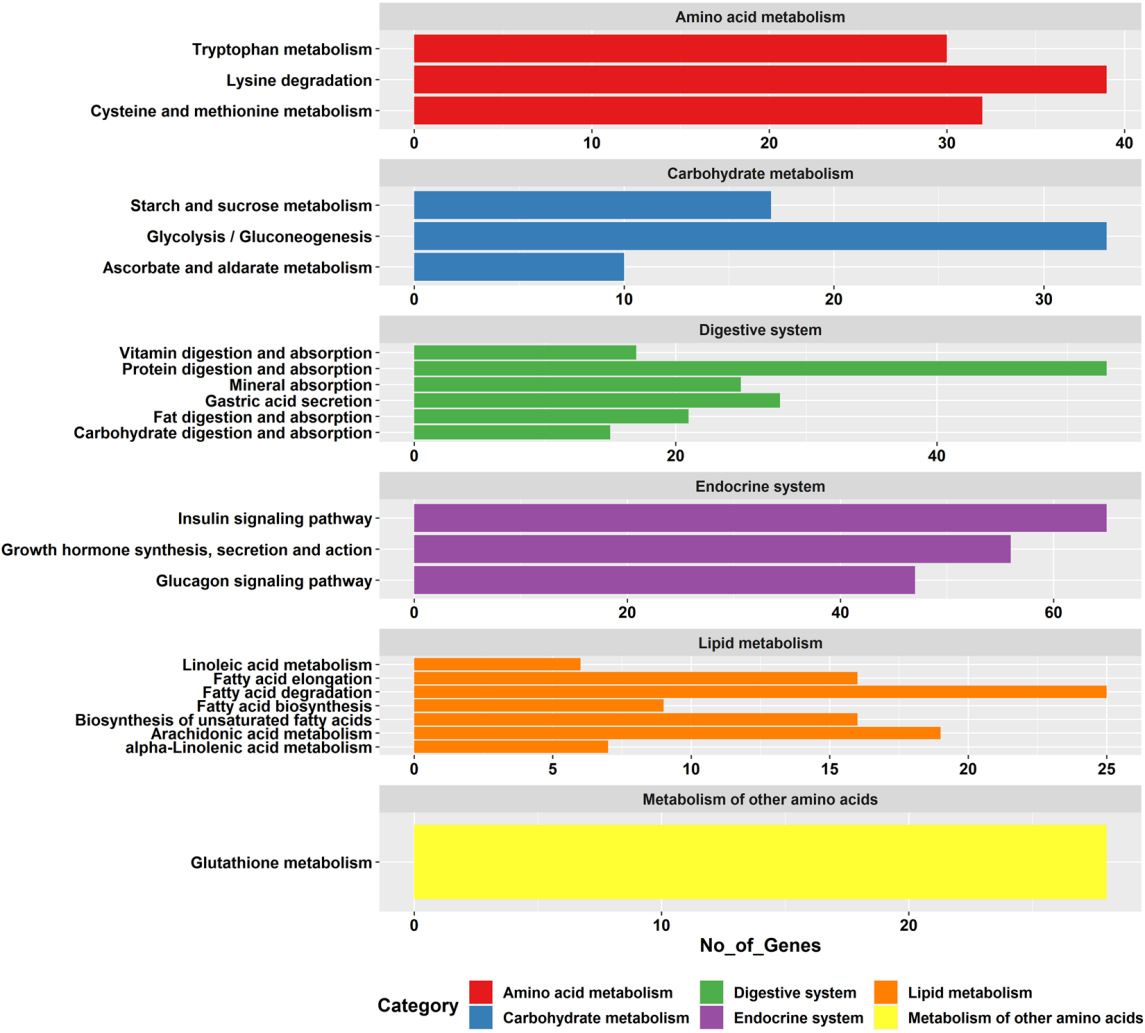
KEGG classification of nutritionally important genes.

**Fig. 6:**
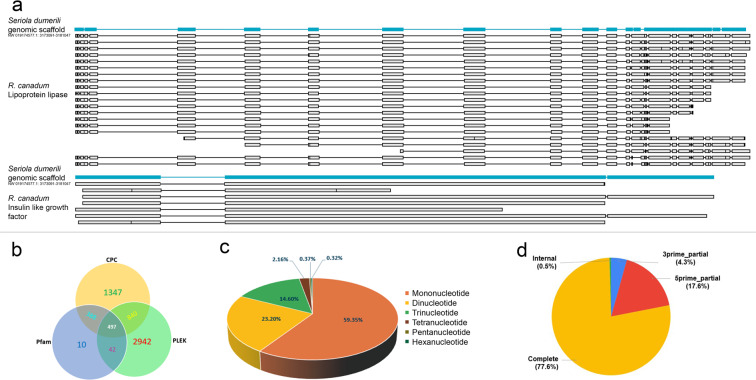
(**a**) The isoforms of lipoprotein lipase and insulin like growth factor_1genes in *R. canadum* showing isoform diversity. **(b)** LncRNA prediction. **(c)** Distribution of microsatellites (SSRs). **(d)** ORF prediction.

Several important functional genes involved in vertebrate nutrition and their isoforms have been identified from functionally annotated transcripts, which can be used in future nutrigenomic studies on cobia. Genes involved in the following biological processes were selected as marker genes: amino acid metabolism, digestive system, lipid metabolism, carbohydrate metabolism, endocrine system and metabolism of other amino acids (Table 3). The KEGG classification of nutritionally important genes is shown in Fig. 5. Of the 129 identified genes of amino acid metabolism, 32 were involved in cysteine and methionine metabolism, 39 in lysine degradation, 28 in glutathione metabolism and 30 in tryptophan metabolism pathways. Under the carbohydrate metabolism pathway, 33 genes were involved in glycolysis/gluconeogenesis, 17 in starch and sucrose metabolism, and 10 in ascorbate and aldarate metabolism. A total of 159 genes involved in the digestive system have been identified, with the following distribution of genes: gastric acid secretion (28), protein digestion and absorption (53), carbohydrate digestion and absorption (15), fat digestion and absorption (21), vitamin digestion and absorption (17) and mineral absorption (25). Among the 168 genes involved in the endocrine system, 56 genes were involved in growth hormone synthesis, secretion and action, 65 in insulin signalling and 47 in glucagon signalling. A total of 98 genes were identified for pathways in lipid metabolism and are distributed as follows: fatty acid biosynthesis (9), fatty acid elongation (16), fatty acid degradation (25), arachidonic acid metabolism (19), linoleic acid metabolism (6), alpha-linolenic acid metabolism (7) and unsaturated fatty acid biosynthesis (16). Isoforms of lipoprotein lipase and insulin- like growth factor genes showing the isoform diversity in full-length transcript data of *R. canadum* is given in Fig. 6a.

**Table 3:** Genes related to nutrition.

KEGG pathway	Metabolism	No. of genes identified
Amino acid metabolism	Cysteine and methionine metabolism	32
	Lysine degradation	39
	Tryptophan metabolism	30
Carbohydrate metabolism	Glycolysis/Gluconeogenesis	33
	Starch and sucrose metabolism	17
	Ascorbate and aldarate metabolism	10
Digestive system	Gastric acid secretion	28
	Protein digestion and absorption	53
	Carbohydrate digestion and absorption	15
	Fat digestion and absorption	21
	Vitamin digestion and absorption	17
	Mineral absorption	25
Endocrine system	Growth hormone synthesis, secretion and action	56
	Insulin signalling pathway	65
	Glucagon signalling pathway	47
Lipid metabolism	Fatty acid biosynthesis	9
	Fatty acid elongation	16
	Fatty acid degradation	25
	Arachidonic acid metabolism	19
	Linoleic acid metabolism	6
	alpha-Linolenic acid metabolism	7
	Biosynthesis of unsaturated fatty acids	16
Metabolism of other amino acids	Glutathione metabolism	28

### Long non-coding RNAs (LncRNAs) prediction

LncRNAs were predicted using three methods including PLEK^41^, Coding Potential Calculator (CPC)^42^ and Pfam structural domain analysis. The common non-coding hits/intersection of the three results were then filtered and considered as LncRNA.

We obtained 4321, 1347 and 937 candidate LncRNAs determined using PLEK, CPC, and Pfam, respectively, and among these 497 (5.97%) were identified in all analyses (Fig. 6b). The length of the LncRNA transcripts ranged from 200 bp to 8198 bp, with a mean length of 1918 bp. The LncRNA results are given in Table 4.

**Table 4:** LncRNA prediction results.

Database	Unshared	Commonly shared	Shared with			Total
			CPC	Pfam	PLEK	
PLEK	2942	497	840	42		4321
CPC	1347	497		388	840	1347
Pfam	10	497	388		42	937

### Detection of Simple sequence repeats (SSRs)

The MISA software (http://pgrc.ipk-gatersleben.de/misa/misa.html) was used to predict the simple repeat markers in the non-redundant reference transcriptome of *R. canadum*, and the minimum repetition time for core-repeat motifs was fixed as follows: 10 for mononucleotides, six for di-nucleotides and five for tri-nucleotides, tetra-nucleotides, penta-nucleotides and hexa-nucleotides. Furthermore, the SSRs were categorized into perfect and complicated (compound or discontinuous) SSRs based on the structural organisation of the repeat motifs.

A total of 35661 transcripts with a total length of 94193725 bp were used for SSR prediction and it was observed that 10449 sequences contained more than one SSR marker. The number of SSRs found in compound formation was 7901, most of which were mononucleotide repeats (25133, 59.35%), dinucleotide repeats (9824, 23.20%), tri-nucleotide repeats (6183, 14.6%), tetra-nucleotide repeats (914, 2.16%), hexa-nucleotide repeats (157, 0.37%) and penta-nucleotide repeats (135, 0.32%). The results of SSR prediction are given in Table 5 and represented in Fig. 6c.

**Table 5:** Number and unit size of SSR identified in the transcriptome.

Total number of sequences inspected	35661
Total size (bp) of examined sequences	94193725
Total number of SSRs identified	42346
Number of sequences containing SSRs	19673
Number of sequences having more than 1 SSR	10449
Number of SSRs involved in compound formation	7901
Unit size and Number of SSRs	
Unit size of SSRs	Number of SSRs
1	25133
2	9824
3	6183
4	914
5	157
6	135

### ORF prediction

In total, 38243 coding sequences were predicted from 35661 transcripts using TransDecoder, with an average length of 448 bp, and there were 2075 transcripts with a length >1000 bp. The coding sequence lengths of ORFs is presented in Fig. 6d.

## Data Records

The raw full-length data (Table 1) were deposited in the NCBI Sequence Read Archive (SRA)^43^ under accession numbers SRR19370125^44^, SRR19370124^45^ and SRR19370123^46^, while the respective BioSamples accession numbers are SAMN28614395, SAMN28614396 and SAMN28614397. Data regarding the identified nutritionally important genes was deposited at the figshare platform^47^. The file contains multiple spreadsheets with the annotated list of genes involved in the metabolism of carbohydrate, protein, lipid, vitamin, mineral, digestive function and bone development in spreadsheets 1 to 7 respectively.

## Technical Validation

**Fig. 7:**
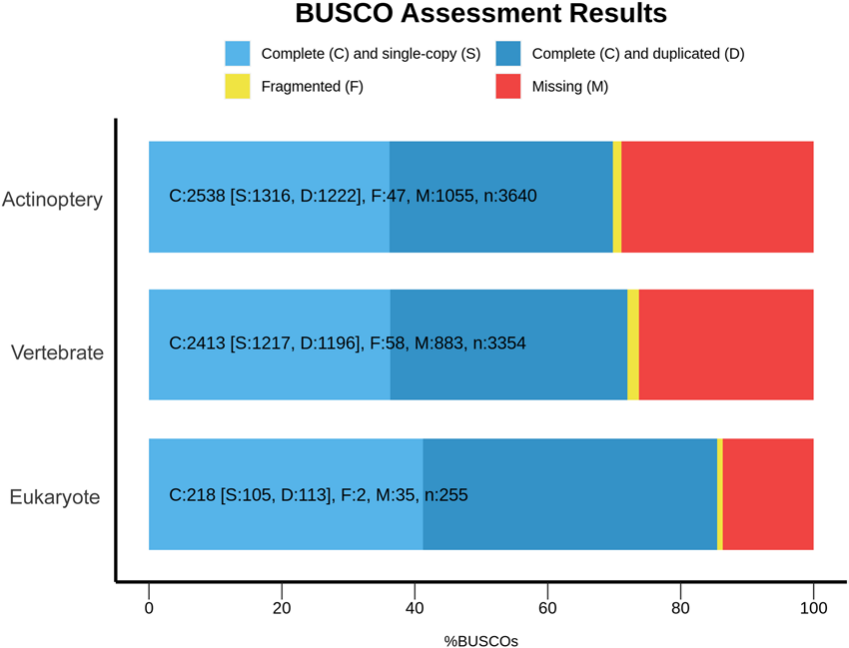
BUSCO analysis results.

The BUSCO analysis results showed that among the 255 conserved eukaryotic orthologous genes, 85.5% complete genes (218 genes) were found in the *R. canadum* transcriptome with an additional 0.78% (2 genes) as fragmented BUSCOs (Fig. 7). Of these, 41.17% were complete single-copy BUSCOs and 5.09% were complete duplicate BUSCOs. A total of 71.94% (2413 genes) of the 3354 orthologues searched for in vertebrates were found in full, with another 1.72% (58 genes) as partial sequences. Of the 3640 orthologues in the eukaryote, 69.72% (2538 genes) were found in full and a further 1.29% (47 genes) as a partial sequence.

## Data Availability

Most of the data analysis was performed using software running on the Linux system, and the version and parameters of the main software tools are described below. (1) SMRTlink: Version 10.1, parameters: No Polish: TRUE, min_zscore: −10 (Default) min_passes 3, Min_predicted_accuracy 0.99. (2) Arrow: parameters: bin_size_kb 1 hq_quiver_min_accuracy 0.99, qv_trim_3p 30, bin_by_primer false. qv_trim_5p (Ignore) qv_trim_3p (Ignore) bin_by_primer false. (3) CD-HIT-Est: Version 4.8.1, parameters: -c 0.96 –n 10 -G 0 - aL 0.00 -aS 0.99. (4) TransDecoder: Version 3.0.1, parameters: -G universal, -m 100. (5) BUSCO: Version 5.3.2, default parameters. (6) BLASTx: Version 2.10.1, parameters: -outfmt 6, -evalue 1e-5. (7) BLASTp: Version 2.10.1, parameters: -outfmt 6, -evalue 1e-5. (8) Metascape: Version 3.5, default parameters. (9) EggNOG: Version 2.1.8, parameters: -m diamond,--itype proteins,--sensmode more-sensitive,--go_evidence non-electronic. (10) PLEK: Version 1.2, parameters: -minlength 200, -isoutmsg 0, -isrmtempfile 1. (11) CPC: Version 2, default parameters. (12) MISA: Version 2.1, default parameters.
